# The cost-effectiveness and consumer acceptability of taxation strategies to reduce rates of overweight and obesity among children in Australia: study protocol

**DOI:** 10.1186/1471-2458-13-1182

**Published:** 2013-12-14

**Authors:** Tracy A Comans, Jennifer A Whitty, Andrew P Hills, Elizabeth Kendall, Erika Turkstra, Louisa G Gordon, Josh M Byrnes, Paul A Scuffham

**Affiliations:** 1 Population and Social Health Research Program, Griffith Health Institute, Griffith University, Brisbane, Australia; 2 Centre for Applied Health Economics, School of Medicine, Griffith University, Brisbane, Australia; 3 Centre for Nutrition and Exercise, Mater Research, Brisbane, Australia; 4 Centre for Musculoskeletal Research, Griffith Health Institute, Griffith University, Brisbane, Australia

## Abstract

**Background:**

Childhood obesity is a recognised public health problem and around 25% of Australian children are overweight or obese. A major contributor is the obesogenic environment which encourages over consumption of energy dense nutrient poor food. Taxation is commonly proposed as a mechanism to reduce consumption of poor food choices and hence reduce rates of obesity and overweight in the community.

**Methods/Design:**

An economic model will be developed to assess the lifetime benefits and costs to a cohort of Australian children by reducing energy dense nutrient poor food consumption through taxation mechanisms. The model inputs will be derived from a series of smaller studies. Food options for taxation will be derived from literature and expert opinion, the acceptability and impact of price changes will be explored through a Citizen’s Jury and a discrete choice experiment and price elasticities will be derived from the discrete choice experiment and consumption data.

**Discussion:**

The health care costs of managing rising levels of obesity are a challenge for all governments. This study will provide a unique contribution to the international knowledge base by engaging a variety of robust research techniques, with a multidisciplinary focus and be responsive to consumers from diverse socio-economic backgrounds.

## Background

Obesity is a major public health issue in the developed world [1] and the rates of childhood obesity are increasing. Australian data from the National Health Survey indicate that the percentages of obesity in 5-12 year-old Australian children rose from 5% in 1995 to 7.8% in 2011-12 [2,3] and 25% of 5-12 year-olds were classified as overweight or obese [2,3]. Children who are obese after age six years have over 50% chance of being obese as an adult compared with a 10% chance for non-obese children [4]. Every organ system can be affected by childhood obesity with hypertension, fatty liver disease, insulin resistance, dyslipidaemia, pulmonary disorders and psychological problems being the most common co-morbidities in children [5,6]. A dose-response relationship exists between duration of obesity for cardiovascular, cancer and all-cause mortality, underlining the importance of targeting the reduction of obesity rates in children and young people [7]. Apart from surgery for adults with a BMI >30 kg/m^2^[8], few interventions for obesity have shown effective long-term outcomes [9,10]. The most promising programs however have been targeted at children [4,11] and may have been more successful in this age group because behaviour is more modifiable [12]. Given that interventions to treat obesity often produce only modest or minimal effects, preventing obesity from occurring in the first place may be far more effective.

Children are major consumers of energy dense nutrient poor “junk foods”. Foods that are energy-dense and nutrient-poor contribute a large proportion (~40%) of the daily energy intake of Australian children [13]. This is despite national guidelines recommending that these foods are only consumed rarely [14].

The steep rise in obesity rates since the 1970s has been blamed by many public health researchers on an increasingly obesogenic environment [15-17]. The relative price of whole foods such as whole milk, fruit and vegetables has risen over the last 20 years whereas that of high-energy nutrient-poor foods such as burgers and fried potatoes has reduced in real terms (taking inflation into account) [18].

In Australia, obese individuals consume 1.8 times more healthcare services per year than is consumed by their normal weight counterparts (an additional $1177 to $2091 per year) [19]. Additional costs attributable to overweight and obesity are accrued for non-healthcare costs and government subsidies resulting in an overall estimated cost of overweight and obesity in Australia in 2005 of AU$21 billion [19].

Reducing consumption of energy dense nutrient poor foods will lead to a reduction in rates of overweight and obesity. An Australian study which modelled the use of a 10% food tax on selected unhealthy food categories found that this strategy could be effective and cost saving in the Australian context [20]. A 20-year US longitudinal study found that a $1.00 increase in price of soda or take away pizza was associated with lower daily energy intake and lower weight [21]. Consequently, taxation that increases the price of junk food is commonly proposed as one mechanism to alter food consumption by discouraging poor food choices.

This paper outlines the protocol of a project that aims to identify the cost-effectiveness and consumer acceptability of taxation strategies to reduce rates of overweight and obesity amongst children in Australia. The specific objectives of this project are to:

1. Identify what types of energy dense nutrient poor foods (hereafter referred to as “junk foods”) would be most likely to result in reduced future rates of obesity in children if consumption of these were reduced.

2. Assess consumer preferences and acceptability of implementing a range of tax-based strategies to reduce junk food consumption in children.

3. Estimate the effect of changes in price of junk foods on the consumption of junk foods and other foods (goods).

4. Model the cost-effectiveness of various taxation strategies in reducing consumption of junk foods, improving health outcomes and reducing future health care costs in children in the medium to long term.

This study has ethical approval from the Griffith University Human Research Ethics Committee: MED/32/12/HREC.

## Methods/Design

**Figure 1:**
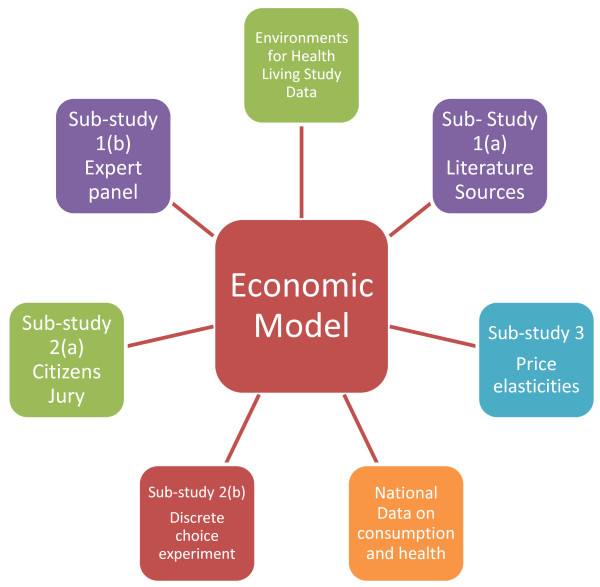
Impact of taxation on obesity rates in children.

This project comprises a series of sub-studies to inform a full economic evaluation of the impact of taxation on obesity rates in children. Figure 1 illustrates the relationship between the sub-studies and the economic model.

For sub-studies one and two (expert panel, citizen’s jury and discrete choice experiment) written informed consent will be obtained from all participants.

**Sub-study 1**: Determine the types of foods contributing to overweight and obesity in children and whether taxation of these is feasible. Two methods will be employed, literature review and expert review.

(a) Two focussed literature reviews will be undertaken. The first literature review will examine food groups considered most important in contributing to childhood obesity, with a specific focus on Australian food intake patterns and also consider individual, household, societal and environmental factors which impact on childhood obesity. The second review will focus on international food taxation strategies on junk food and their effectiveness.

(b) An expert panel on nutrition will be convened to assess the evidence collated from the literature reviews and provide expert opinion on the robustness of the evidence, the comprehensiveness of the foods identified in the first review and whether any gaps exist in the literature reviews which should be supplemented from other sources. A nominal group technique [22] will be used to gain consensus on the most important foods contributing to childhood obesity and what the best targets for taxation would be. The nominal group technique enables effective group decision making by ranking priorities and by undertaking a discussion of the topic moderated by a facilitator [23].

**Sub-study 2**: Assess the consumer acceptability and societal preferences for implementing a range of tax-based strategies to reduce junk food consumption in children. A mixed methods approach will be employed; triangulation of these two methods will strengthen the robustness of the research findings:

(a) A Citizens’ Jury will be held with approximately 12 members of the general public, with a specific focus on exploring the acceptability of preventative strategies, especially tax-based strategies, identified as having the potential to reduce childhood obesity. The Citizens’ Jury is a deliberative method of engaging the public in policy issues. Participants will be presented with a dilemma regarding the prevention of obesity in children, and members of the expert panel convened for Aim 1 will present evidence and be cross-examined by the participants (“jurors”). Citizens’ juries are modelled on the legal jury system where a selection of citizens come together to hear evidence, discuss the issues and make deliberations [24,25]. The citizens are taken to be a fair representation of the views and opinions of the general public. Previous work indicates jurors engage fully in this process, carefully consider the evidence, express their views and develop community and altruistic views toward the issues [26,27]. A combined random/purposive sampling approach will be employed for the Citizens’ Jury to ensure an unbiased selection of diverse participants, with potential participants selected from the electoral roles in Queensland. Data will be analysed thematically using a systematic approach.

(b) A discrete choice experiment will be undertaken in parents of young children to quantify preferences and trade-offs around tax-based strategies aimed at reducing the consumption of selected junk foods by children [28]. In the discrete choice experiment, respondents will be given a number of hypothetical choice scenarios and asked to indicate their preference between competing alternatives. Each scenario will be described by a number of attributes (e.g. price, convenience of purchase, nutritional content, “traffic light” labelling [29]. The specific foods or combinations of foods to be considered, and the attributes and levels used to describe them (e.g. price levels, present/absent) will be developed from the literature reviews in Aim 1 and from the Citizens’ Jury. The levels of the attributes will be varied between alternatives according to a systematic design that optimises the efficiency of the preference estimates obtained from a regression model of the choice data, such as a multinomial logit model or its more generalised forms [25]. The parameter estimates from the model indicate the relative importance of different attribute levels to the decision and the trade-offs individuals are willing to make between them. The discrete choice experiment is an established method for quantifying consumer preferences [28], and will identify priorities for implementing tax-based strategies to prevent childhood obesity from the perspective of consumer acceptability and likelihood of preference shift. It will also estimate the sensitivity of choice to price (to inform Substudy 3).

Participants for the discrete choice experiment will be parents of children, since they make food choices on behalf of their children. Parents will be recruited from the existing Environments for Health Living (EFHL) longitudinal study of infants born in areas of South East Queensland and Northern NSW [30]. The community in the EFHL study has one of the fastest population growths in Australia and contains sizeable areas of low socio-economic status and distinct ethnic groups with almost 30% of mothers born overseas [31]. Previous overseas and Australian research has demonstrated clear links between socio-economic status and obesity [32,33]. Therefore, the cohort in the EFHL study represents an ideal group with which to conduct choice experiments and analyse rates and trends of childhood obesity and adult obesity within families.

The EFHL study commenced in 2006 with annual waves of recruitment; at the end of 2011, 3,368 mother and infant dyads have been recruited [31]. For the discrete choice experiment, simple random sampling will be used to select up to 1200 mothers from across the waves of recruitment to capture mothers of children aged 0-5 years. These mothers and their partners will be invited to complete the discrete choice experiment, giving an estimated completed sample size of >900 (assuming 50% of mothers and 25% of partners complete a discrete choice experiment). Samples used in previous discrete choice experiments confirm that 900 is ample to provide precise preference estimates, including subgroup analyses using covariates to explore the impact of participant characteristics (e.g. age, gender, education, income, number of children) on preferences [34].

**Sub-study 3**: Estimate the response to changes in the price of junk food on the consumption of junk food and other foods by children in Australia.

Two approaches will be used to achieve this aim with the price and income elasticities estimated from both methods employed in the base case and sensitivity analyses of the economic models:

(a) Price elasticities, using data from household expenditure surveys, will be estimated using regression analysis of the percentage change in consumption and the percentage change in the price of that food product (own-price elasticity) and a percentage change in the price of other foods (cross-price elasticity), taking into account socio-economic factors (for example household income). In order to estimate the impact of price changes specific to children’s consumption, this analysis will use data from the Household Expenditure Survey 2011 [35].

(b) Contemporaneously, price and cross-price elasticities for selected foods adjusted for income will be estimated from the discrete choice experiment, by including price and/or taxation level as an attribute in the discrete choice experiment design and estimating the impact of a 1% change in price on the probability of choice for a selected food alternative [34,36]. Estimates derived from the above approaches will be compared to international estimates, where available.

**Sub-study 4**: Development of an economic model to measure the costs and benefits of food taxation to address childhood levels of obesity.

A population simulation model will be developed to determine the potential lifetime benefits of reducing the prevalence of obesity in children in Australia by use of the taxation strategies identified in the first three steps of the project. The time horizon for the model will be lifetime with sub-analyses conducted at five year intervals. Costs and benefits will be discounted by 3% according to the most commonly used rate in Australia with varying rates used in a sensitivity analysis [37].

**Data Sources**: As well as data derived from the study and from the EFHL cohort, other demographic characteristics, health status and prevalence estimates of obesity will be obtained from the 2011-12 Australian National Health Survey Australian data [3]. Health care usage will be collected from a nested case-control study within the EFHL cohort that includes inpatient hospitalisations, emergency department visits, Medicare data for GP consultations and all prescribed medications. Overweight and obesity in children will be defined using an internationally recognised definition [38,39]. Using the data collected through the EFHL study, comparisons of health service usage between overweight and obese and normal weight children will be undertaken correcting for covariates such as socio-economic status.

The resultant model will provide estimates of the likely costs and benefits of implementing taxation as a strategy to prevent obesity. Varying the parameters in the model will provide predictive information on where the levels of taxation and regulation should lie for efficient use of resources and to maximise the benefits achieved for a given cost.

## Discussion

Using taxation as a strategy to reduce consumption of junk foods is more complex than taxing alcohol or cigarettes as there is no clear definition of what constitutes healthy or harmful food. As a result, it has been relatively easy for opponents of taxation to argue against having differential taxation rates for different food types, relegating the strategy to the “too hard” basket. The implementation of food taxes in 'real life’ settings is limited and the analysis of the effectiveness of these is even more limited. Many states in the U.S have implemented taxes on sugar sweetened beverages, and France implemented a tax on drinks containing added sugar or sweetener in 2012. Denmark introduced a 'fat tax’ in 2011 that was repealed after one year; and various other countries have *ad hoc* implementation of taxes on unhealthy foods. However, the purpose of these taxes has generally been to raise revenue, rather than influence consumption [40]. California introduced a snack food tax in 1991 that resulted in a 10% drop in sales and Maryland levied a 5% tax on snack food in 1992 with one snack food manufacturer noting $500,000 in lost sales [41]. Despite these taxes being successful in the aim of reducing snack food consumption, they were repealed due to intense lobbying by the food industry and arguments that they were arbitrary, confusing and regressive [42].

Research examining the relationship (elasticity) between food prices and intake is scarce; however, inverse associations have been found suggesting that increasing (or differential) taxation rates might be an effective anti-obesity strategy. To date, price elasticity estimates for Australia have not focused on 'junk food’ but rather, food as an aggregated product [43]. Estimates are only available in broad categories of food products such as dairy, meat, fruits and vegetables [27], or specific items within a food category such as beef, lamb and pork within the meat category [28]. In addition, this work has not focussed on the impact of changes in price in the food intake of children.

This proposed research, through its comprehensive assessment of the literature, inclusion of public preferences and economic modelling, will identify the most effective and acceptable approach to implement a taxation on energy dense nutrient poor foods in the Australian context. It is intended that the results of the study will be easily interpreted and translated to policy and practice. This study is unique in that it will identify what strategies are likely to be effective and what conditions will be acceptable to the public in the fight against obesity. In Australia, the National Health and Hospitals Reform Commission has recently recommended that a systematic mechanism be developed to formulate health care priorities in a way that incorporates community perspectives as well as economic and clinical considerations [32]. The challenge of how to effectively gain community perspectives is a crucial consideration in public policy at the moment, and is addressed by this study. To guide the feasibility and successful implementation of effective population based approaches in the prevention of obesity in Australia, it is essential to gather information about how consumers will respond to large scale, yet sensitive, reforms.

The methods proposed to assess consumer acceptability are innovative. The Citizens’ Jury approach is becoming an increasingly popular and effective method to engage consumers in matters of public policy. Although the discrete choice experiment is an established method for quantifying consumer preferences and willingness to pay for health [28], to our knowledge it has not previously been applied to assess the impact of public health strategies on junk food consumption, nor to estimate price elasticity in any health context. The use of discrete choice experiments to establish context specific parameters around taxation will inform the development of economic models in this area in the future.

Taxation may only be successful however if other important conditions are met. For instance, the authors of “Food fight” argue that for a food tax to be successful, the aim to decrease consumption of unhealthy foods must be explicit [42]. In addition, revenue generated must be allocated to initiatives that are publically supported and the way taxes are applied must be less arbitrary, easy to understand and undertaken with maximum health benefit as the goal [42]. The key point is that the tax must be acceptable to consumers and that revenue raised should be directed to improving public health and nutrition [44].

Like many countries around the world experiencing rapidly rising obesity rates in their populations and obesegenic environments, the Australian government is looking for innovative ways to address this serious health problem from a population health perspective. Systemic approaches such as taxation on junk food have been found to have mixed results to date, in part, due to being quickly implemented without a strong evidence-base for planning and little understanding of the population impact. This study will provide a unique contribution to the international knowledge base by engaging a variety of robust research techniques, with a multidisciplinary focus and be responsive to consumers from diverse socio-economic backgrounds. This research is comprehensive and will deliver a policy-driven response to directly influence health reform aiming to reduce obesity in Australia and elsewhere.

## Abbreviations

EFHL: Environments for health living study.

## Competing interests

The authors declare that they have no competing interests.

## Authors’ contributions

TC conceived the overall study and drafted the manuscript. JW designed the discrete choice experiment. JB drafted the price elasticities section. LG assisted in design of the economic modelling. AH, ET, EK and PS participated in the design of the study. All authors read and approved the final manuscript.

## Pre-publication history

The pre-publication history for this paper can be accessed here:

http://www.biomedcentral.com/1471-2458/13/1182/prepub
